# Brain microstructural and metabolic alterations detected *in vivo* at onset of the first demyelinating event

**DOI:** 10.1093/brain/awab043

**Published:** 2021-04-27

**Authors:** Sara Collorone, Ferran Prados, Baris Kanber, Niamh M Cawley, Carmen Tur, Francesco Grussu, Bhavana S Solanky, Marios Yiannakas, Indran Davagnanam, Claudia A M Gandini Wheeler-Kingshott, Frederik Barkhof, Olga Ciccarelli, Ahmed T Toosy

**Affiliations:** 1 NMR Research Unit, Queen Square MS Centre, Department of Neuroinflammation, UCL Queen Square Institute of Neurology, Faculty of Brain Sciences, University College London, London, UK; 2 Centre for Medical Image Computing (CMIC), Department of Medical Physics and Biomedical Engineering, University College London, London, UK; 3 Universitat Oberta de Catalunya, Barcelona, Spain; 4 Centre for Medical Image Computing (CMIC), Department of Computer Sciences, University College London, London, UK; 5 Department of Brain Repair and Rehabilitation, University College London Institute of Neurology, Faculty of Brain Sciences, UCL, London, UK; 6 Department of Brain and Behavioral Sciences, University of Pavia, Pavia, Italy; 7 Brain MRI 3T Research Centre, IRCCS Mondino Foundation, Pavia, Italy; 8 Department of Radiology and Nuclear Medicine, Amsterdam University Medical Centers, Vrije Universiteit, The Netherlands; 9 National Institute for Health Research, University College London Hospitals, Biomedical Research Centre, London, UK

**Keywords:** multiple sclerosis, relapsing-remitting, sodium magnetic resonance imaging, diffusion magnetic resonance imaging, NODDI, axonal injury

## Abstract

In early multiple sclerosis, a clearer understanding of normal-brain tissue microstructural and metabolic abnormalities will provide valuable insights into its pathophysiology. We used multi-parametric quantitative MRI to detect alterations in brain tissues of patients with their first demyelinating episode. We acquired neurite orientation dispersion and density imaging [to investigate morphology of neurites (dendrites and axons)] and ^23^Na MRI (to estimate total sodium concentration, a reflection of underlying changes in metabolic function). In this cross-sectional study, we enrolled 42 patients diagnosed with clinically isolated syndrome or multiple sclerosis within 3 months of their first demyelinating event and 16 healthy controls. Physical and cognitive scales were assessed. At 3 T, we acquired brain and spinal cord structural scans, and neurite orientation dispersion and density imaging. Thirty-two patients and 13 healthy controls also underwent brain ^23^Na MRI. We measured neurite density and orientation dispersion indices and total sodium concentration in brain normal-appearing white matter, white matter lesions, and grey matter. We used linear regression models (adjusting for brain parenchymal fraction and lesion load) and Spearman correlation tests (significance level *P* ≤ 0.01). Patients showed higher orientation dispersion index in normal-appearing white matter, including the corpus callosum, where they also showed lower neurite density index and higher total sodium concentration, compared with healthy controls. In grey matter, compared with healthy controls, patients demonstrated: lower orientation dispersion index in frontal, parietal and temporal cortices; lower neurite density index in parietal, temporal and occipital cortices; and higher total sodium concentration in limbic and frontal cortices. Brain volumes did not differ between patients and controls. In patients, higher orientation dispersion index in corpus callosum was associated with worse performance on timed walk test (*P*** ***=*** **0.009, B = 0.01, 99% confidence interval = 0.0001 to 0.02), independent of brain and lesion volumes. Higher total sodium concentration in left frontal middle gyrus was associated with higher disability on Expanded Disability Status Scale (*r*_s_ = 0.5, *P*** ***=*** **0.005). Increased axonal dispersion was found in normal-appearing white matter, particularly corpus callosum, where there was also axonal degeneration and total sodium accumulation. The association between increased axonal dispersion in the corpus callosum and worse walking performance implies that morphological and metabolic alterations in this structure could mechanistically contribute to disability in multiple sclerosis. As brain volumes were neither altered nor related to disability in patients, our findings suggest that these two advanced MRI techniques are more sensitive at detecting clinically relevant pathology in early multiple sclerosis.

## Teaser

Using a multi-parametric quantitative MRI approach, Collorone *et al.* reveal microstructural and metabolic alterations in the brains of patients at the onset of clinically isolated syndrome or multiple sclerosis. The changes were associated with disability outcomes, and were not captured by conventional MRI.

## Introduction

In multiple sclerosis, diffuse pathological changes affect brain tissues that exhibit normal appearances with conventional MRI.^1^ Post-mortem studies have characterized these processes in advanced multiple sclerosis,^2^ but these studies are understandably rare in early patients, with very small cohorts and often atypical cases.^3^

Quantitative MRI techniques can measure, *in vivo*, microstructural changes, and contribute to our understanding of multiple sclerosis from its early phases.^4^ Furthermore, when metabolic quantitative MRI is employed, further insights into causative mechanisms of these changes can be probed.^5^ However, multi-parametric approaches including structural and metabolic quantitative MRI are rare and have not yet been performed in early patients, except for the study of specific brain structures.^6^^,^^7^

In this study, we investigated microstructural and metabolic alterations in the brains of patients at the onset of their first neurological episode suggestive of demyelination. We combined, for the first time, two quantitative MRI techniques: neurite orientation dispersion and density imaging (NODDI)^8^ and ^23^Na MRI.^9^ Used independently, they have contributed promising results in multiple sclerosis, but have never been used at the early stage of the condition, after the first clinical symptoms.

NODDI, a multi-compartmental diffusion MRI technique, provides two voxelwise metrics of neurite morphology: the neurite density index (NDI) and the neurite orientation dispersion index (ODI), unconfounded by CSF contamination. NDI estimates the fraction of axons and dendrites within the neural tissue: being primarily sensitive to neuronal and axonal density.^10^ NDI is considered a marker of neuroaxonal damage. ODI quantifies the variability of neurite orientations—increases in the crossing or fanning of axons increase ODI.^11^ In established multiple sclerosis, studies have shown reduced NDI in both white matter lesions and the normal-appearing white matter.^12–15^ Furthermore, different patterns of NDI and ODI alterations exist in specific white matter and grey matter areas, showing clinical relevance.^13^^,^^15^

^23^Na MRI measures the total voxelwise sodium concentration (TSC). Researchers have associated metabolic changes in TSC with neuroaxonal dysfunction and loss. Studies have shown that electrophysiological alterations of demyelinated axons^16^ and mitochondrial dysfunction^17^^,^^18^ lead to intracellular sodium accumulation in multiple sclerosis^19^ that can result in TSC increase. In the presence of oedema or neuroaxonal loss, the expansion of the extracellular space, where sodium is abundant, can also increase the TSC. The application of ^23^Na MRI to multiple sclerosis cohorts showed that, in normal-appearing white matter, white matter lesions and grey matter, TSC was increased compared with healthy controls^20–24^ and this was associated with increased disability.^21–24^ A recent study in a cohort of patients assessed 15 years after clinically isolated syndrome (CIS) showed that TSC in the white matter and grey matter was higher in patients who had developed multiple sclerosis than in patients who had remained CIS and in healthy controls.^25^

In CIS patients within 6 months from onset, previous studies using diffusion tensor imaging have demonstrated alterations in the normal-appearing white matter, suggesting tissue damage^7^^,^^26^^,^^27^ although over time they did not correlate with lesion accumulation,^28^ relapse rate,^29^ or multiple sclerosis conversion.^30^ The diffusion tensor model does not suffice to characterize fully the complexity of water diffusion in biological media, and therefore lacks the sensitivity and specificity to accurately determine subtle pathological changes as those taking place in multiple sclerosis.^31^ Furthermore, in CNS areas characterized by low anisotropy, such as grey matter, the diffusion tensor model does not adequately describe the tissue microstructure.^32^ The NODDI model is one of several potential alternative approaches that aim to overcome these limitations.^31^^,^^33^

Previous studies have analysed metabolic alterations in early patients using magnetic resonance spectroscopy (MRS). Because of the limitations of MRS, some studies only analysed a single voxel^34^ or a small brain area.^7^ Other studies found increased whole-brain *N*-acetylaspartate, a marker of neuro-axonal loss, in CIS patients^35–38^ more prominent in patients converting to multiple sclerosis.^36^^,^^37^^,^^39^ Since multiple sclerosis can be considered a channelopathy,^40^ 23Na MRI can give insights into multiple sclerosis-specific pathological mechanisms underlying neurodegeneration.

Combining NODDI with ^23^Na MRI provides complementary voxelwise information related to potential brain abnormalities and may address questions about early multiple sclerosis pathogenesis. In multiple sclerosis, increases in TSC can reflect both high intracellular sodium concentration, a sign of neuro-axonal dysfunction, as well as increased extracellular space, as for neuro-axonal loss or oedema.^23^ Hence, by using NODDI and ^23^Na MRI, we can document brain areas with increased TSC and reduced NDI, suggesting neuro-axonal loss, or with isolated TSC increase, suggesting initial neuroaxonal dysfunction^41^ or functional changes.^42^

The goal of this study was to demonstrate that a multi-parametric quantitative MRI approach can detect alterations in the brain tissues of early CIS and multiple sclerosis patients not captured by conventional MRI. The specific aims were: (i) to quantify NDI, ODI and TSC in normal-appearing white, white matter lesions, and grey matter; and (ii) to determine if microstructural and metabolic alterations were associated with clinical outcomes.

## Materials and methods

### Participants

We enrolled 42 patients within 3 months of the onset of neurological symptoms suggestive of their first demyelinating episode from the National Hospital of Neurology and Neurosurgery and Moorfields Eye Hospital, London, UK. We also recruited 16 age- and sex-matched healthy controls.

Inclusion criteria were age between 18 and 65 years, and the ability to give written informed consent in English and to have an MRI scan (assessed by passing an MRI safety checklist). Exclusion criteria were: a history of past neurological episodes, the presence of antibodies against aquaporin-4 or myelin oligodendrocyte glycoprotein, which was routinely assessed in patients with optic neuritis, and other medical conditions potentially affecting the CNS.

The local ethical committee approved the study protocol and all subjects gave written informed consent (Study Ref: 13/LO/1762; 13/0231-CIS2013).

### Clinical assessments

In patients, we evaluated physical disability using the Kurtzke Expanded Disability Status Scale (EDSS),^43^ the Timed 25-Foot Walk Test for lower limb function, and, the 9-Hole Peg Test for upper limb function.^44^

We assessed cognition in patients using the Paced Auditory Serial Addition Test (PASAT), for auditory processing speed and attention,^44^ the Symbol Digit Modalities Test (SDMT) for visual information processing speed, the California Verbal Learning Test-II (CVLT-II) for verbal memory, and the Brief Visuospatial Memory Test-Revised (BVMT-R) for visuospatial memory.^45^

We recorded the years of education for each patient and calculated *z*-scores from their cognitive tests using the Brief Cognitive Assessment for Multiple Sclerosis (BICAMS) initiative dataset (https://www.bicams.net)^46^ and, for the PASAT, the National Multiple Sclerosis Society Task Force database.^44^

### MRI acquisition

We used a 3 T Achieva MRI scanner (Philips Medical Systems) with a 32-channel head coil and a maximum gradient strength of 62 mT.m^−1^.

All participants underwent structural MRI of the brain and spinal cord. The brain MRI protocol included: (i) axial proton density/T_2_-weighted imaging using a 2D dual-echo turbo spin echo sequence (repetition time = 3500 ms; echo time = 19/85 ms; field of view = 240 × 180 mm^2^; voxel size = 1× 1 × 3 mm^3^); (ii) 3D T_1_-weighted turbo field echo (repetition time = 6.9 ms; echo time = 3.1 ms; inversion time = 821 ms; field of view = 256 × 256 mm^2^; voxel size = 1× 1 × 1 mm^3^); (iii) 3D FLAIR (repetition time = 8000 ms; echo time = 388 ms; inversion time = 2400 ms; field of view = 250 × 250 mm^2^; voxel size = 1.2 × 1.2 × 1.2 mm^3^); (iv) for patients only, axial pre- and post-gadolinium T_1_-weighted turbo spin-echo sequences (repetition time = 625 ms; echo time = 10 ms; field of view = 240 ×180 mm^2^; voxel size = 1× 1 × 3 mm^3^). The spinal cord MRI protocol included: (i) sagittal proton density/T_2_-weighted images (repetition time = 3500 ms; echo time = 22/78 ms; voxel size = 1 × 1.8 × 3 mm^3^); (ii) for patients only, pre- and post-gadolinium sagittal T_1_-weighted turbo spin-echo sequences (repetition time = 600 ms; echo time = 8 ms; voxel size = 1 × 1.8 × 3 mm^3^).

We acquired brain multi-shell diffusion-weighted images using a 2D echo-planar imaging sequence (repetition time: 12 000 ms; echo time: 91 ms; field-of-view: 192 × 222 mm^2^ voxel size = 2.5 × 2.5 × 2.5 mm^3^) with the following parameters: b = 300 s/mm^2^, eight directions; b = 711 s/mm^2^, 15 directions; b = 2000 s/mm^2^, 30 directions; and eight interleaved non-diffusion-weighted (b = 0) images.

Unfortunately, ^23^Na MRI underwent maintenance procedures that could not be postponed and 32 of 42 patients, and 13 of 16 healthy controls underwent ^23^Na MRI. Using a single tuned ^23^Na coil (RAPID BioMed), we acquired ^23^Na images (repetition time: 120 ms; echo time: 0.27 ms; field of view: 240 × 200 mm^2^; voxel size = 3 × 3 × 3 mm^3^). For TSC quantification, calibration phantoms of 80 mM and 40 mM sodium concentration were fixed to the subject’s head, in the field of view, during the scan.

We also acquired a ^1^H T_2_-weighted dual-echo scan (repetition time: 3875 ms; echo times: 19/85 ms), with the same spatial resolution of the ^23^Na sequence, whilst the subject was in the sodium coil, using the scanner body coil. We used this T_2_-weighted image for the sodium imaging post-processing.

### MRI post-processing

**Figure 1:**
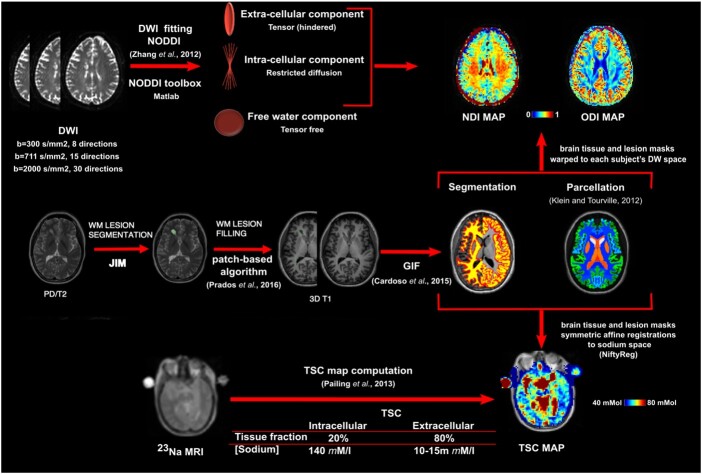
**MRI post-processing.** MRI post-processing for lesion masks, NODDI and ^23^Na MRI (images from a patient presented in the native space for each modality). DWI = diffusion-weighted imaging; GIF = geodesic information flow; PD = proton density; WM = white matter.

Figure 1 summarizes the MRI post-processing workflow. We outlined the T_2_-hyperintense lesions in the white matter of each patient on the 2D proton density/T_2_-weighted image using a semi-automated edge finding tool in JIM v6.0 (Xinapse systems; http://www.xinapse.com/). Using the pre- and post-gadolinium T_1_-weighted sequences, we also outlined the T_1_-hypointense lesions and gadolinium-enhancing lesions. Lesion masks were checked against the 3D FLAIR sequence and corrected through interobserver-agreement by two experienced raters (S.C. and I.D.). Subsequently, we computed the volume of the T_2_-hyperintense and T_1_-hypointense (non-enhancing) lesions for each subject.

Two experienced raters (F.B. and I.D.) assessed white matter lesion number, location and contrast-enhancement and, subsequently, determined the CIS or multiple sclerosis diagnosis according to the revised 2017 McDonald criteria.^47^ For this study, patients were recruited from June 2014 to May 2018: only four were recruited after the publication of the 2017 revision of the McDonald criteria in February 2018. Nevertheless, we used this latest revision for the multiple sclerosis diagnosis to allow comparisons with current scientific literature and because, for the aims of this cross-sectional study, we did not divide patients according to CIS and multiple sclerosis diagnosis: patients were included in our analysis as a single category.

Then, proton density/T_2_- and 3D T_1_-weighted images were rigidly registered and lesion masks were resampled in 3D T_1_ space. Subsequently, we filled the 3D T_1_-weighted images using a non-local patch-match lesion filling algorithm.^48^ The filled 3D T_1_-weighted images were then parcellated and segmented into grey matter (cortical and deep grey matter) and white matter using Geodesic Information Flows (GIF)^49^ method v3.0^50^ (http://niftyweb.cs.ucl.ac.uk) following the Desikan-Killiany-Tourville brain parcellation protocol^51^ (Supplementary Table 1). Afterwards, we obtained, for each subject, volumes and fractions of the grey matter and normal-appearing white matter. All segmentations were quality checked. Both NODDI and ^23^Na MRI have low spatial resolution (2.5 mm and 3.0 mm isotropic voxels, respectively). Therefore, for the evaluation of NDI, ODI and TSC in the grey matter areas, we grouped together areas with anatomical proximity and functional similarity, as shown in Supplementary Table 1, and we also computed the volumes of these areas.

Each diffusion-weighted image was corrected for eddy current-induced distortions and subject motion using ‘eddy’ in FSL 6.0 (FMRIB, Oxford, UK)^52^ and the original (not lesion-filled) 3D T_1_-weighted image was co-registered to the mean b = 0 image using NiftyReg software package (http://niftyreg.sf.net). All undistorted diffusion-weighted data, as well as their anatomical 3D T_1_ alignment, were quality checked. For NODDI fitting, the MATLAB (The MathWorks, Inc., Natick, Massachusetts, USA) NODDI toolbox (http://nitrc.org/projects/noddi_toolbox) using default settings was used to generate ODI and NDI maps.^8^ We then warped the brain tissue masks, as well as the lesion masks for patients, to each subject’s diffusion-weighted space to allow individual characterization of microstructural properties in normal-appearing white and grey matter compartments and in white matter lesions.

NODDI is a multi-compartment technique that models different signal sources within the same imaging voxel. By construction, NODDI metrics account for partial volume effects within imaged voxels. A clear example of this is NODDI isotropic volume fraction, which is designed to capture the amount of free water (i.e. the CSF) in each voxel.^8^

For each subject, we quality checked the brain masks: if necessary, they were manually corrected and ODI and NDI recomputed; if this was unsuccessful, then that subject’s brain area was discarded from further analysis.

TSC was quantified voxelwise using a linear method, dependent on the calibration phantoms. Using NiftyReg software package, a set of symmetric affine registrations were computed and concatenated to transform tissue and lesion masks from 3D T_1_-weighted to TSC space (for more details see)^22^^,^^25^ We used an automated voxel-by-voxel partition-based correction method that removed the contribution of CSF sodium from TSC maps in native sodium space.^22^ Brain tissue masks, and lesion masks for patients, were used to obtain TSC in normal-appearing white matter, white matter lesions, and grey matter. For each subject, we quality checked the TSC maps.

### Statistical analysis

Group differences in demographic characteristics were assessed using two-sample *t*-tests for continuous variables and chi-square tests for categorical variables.

Differences between patients and controls in brain parenchymal fraction, normal-appearing white matter and global grey matter volumes were assessed with linear regression, correcting for age and sex. Using the same model, we assessed the differences between patients and controls in the volume of the grey matter areas parcellated with GIF. Subsequently, group differences in NODDI metrics and TSC in the different brain areas were assessed with linear regression, adjusting for age and sex. For grey matter, the corresponding volume of the parcellated area was added to the model as a predictor, if it was found significantly different between patients and controls.

To overcome the assessment of normality assumption for each area, we used bootstrapping with 1000 repetitions.

In patients, we computed the differences in NDI, ODI and TSC between white matter lesions (all T_2_-hyperintense, T_1_-hypointense, and gadolinium-enhancing) and normal-appearing white matter with paired *t-*tests.

In patients, after identifying the areas where NDI, ODI and TSC were significantly different from healthy controls, we assessed their relationships with lesional and clinical variables. This was to focus our hypotheses on brain regions more likely to exhibit clinically meaningful associations and to help reduce the number of extraneous statistical tests.

Since lesion volumes have a skewed distribution, we log-transformed T_2_-hyperintense and T_1_-hypointense lesion volumes to allow parametric testing.^53^ Then, we used linear regression with bootstrapping to assess the relationship between NDI, ODI and TSC in brain areas with lesion volumes as well as with NDI, ODI and TSC in the T_2_-hyperintense lesions.

For the Timed 25-Foot Walk and the 9-Hole Peg Test, we used linear regression to assess associations between altered NDI, ODI and TSC and these metrics. Age and sex were kept as covariates in the model if significantly associated with these clinical variables. For cognitive tests, we used the *z*-scores adjusted for age, sex and education level. In case of significant associations between NDI, ODI and TSC and disability scores, we reran the regressions, entering brain parenchymal fraction and log-transformed T_2_-hyperintense lesion volume as independent variables to test the influence of conventional MRI metrics on our model.

Since the EDSS score is not normally distributed, we used the Spearman’s rho correlation coefficient for the associations between NDI, ODI and TSC and EDSS scores.

Because of the exploratory nature of the study, we did not perform correction for multiple comparisons, but, for robust regression estimates, we used a significance level of 1% and provide 99% confidence intervals (CIs).

We performed statistical analyses with Stata v. 14.1 (Stata Corporation, College Station, Texas, USA).

### Data availability

Raw data were generated at NMR Unit (UCL). Derived data supporting the findings of this study are available from the corresponding author on request.

## Results

The brain tissue volumes, demographic and clinical characteristics of the participants are shown in Table 1. After quality checking, we discarded two patients from the ^23^Na MRI (failure of the calibration phantoms) and two patients from the NODDI analyses (movement artefacts).

**Table 1:** Brain tissue volumes, demographic and clinical characteristics of patients and healthy controls

	**Patients** **(*n *=* *42)**	**Healthy controls** **(*n *=* *16)**	*P*-values
Age, years	33.1 ± 1.03	33.3 ± 1.7	0.89^a^
Sex, female: male (% female)	23:19 (55)	9:7 (56)	0.92^b^
Syndrome, *n* (%)			
Optic neuritis	35 (83)	–	
Myelitis	2 (5)	–	
Brainstem syndrome	2 (5)	–	
Supratentorial syndrome	2 (5)	–	
Multifocal	1 (2)		
Steroids, *n* (%)	10 (24)	–	–
Months from onset	2.1 ± 1.2		
Grey matter volume, ml	670.2 ± 52	670.8 ± 66.2	0.97^c^
White matter volume, ml	454.7 ± 39.6	467 ± 50.6	0.33^c^
Brain parenchymal fraction	0.76 ± 0.009	0.76 ± 0.02	0.64^c^
White matter T_2_-hyperintense lesion volume, ml, median (range)	3.2 (0–42.2)	–	
White matter T_1_-hypointense lesion volume, ml, median (range)	0.3 (0–6.2)	–	
Spinal cord lesions, median (range)	0 (0–7)		

Data are presented as mean ± standard deviation, unless otherwise indicated.

a Two-sample *t*-tests.

b Pearson's chi-squared test.

c Linear regression model correcting for age and sex.

Patients and healthy controls did not differ significantly in terms of age, sex, brain parenchymal fraction, white matter and grey matter volume (Table 1), and for parcellated grey matter volumes.

Thirty-six patients had T_2_-hyperintense white matter lesions, 22 of whom also had T_1_-hypointense lesions and 13 had gadolinium-enhancing lesions. Thirty patients had dissemination in space and nine of them also fulfilled the 2017 McDonald criteria for multiple sclerosis with additional dissemination in time.^47^

Table 2 reports the clinical scores of the cohort. Table 3 reports the significant (*P* ≤ 0.01) results in normal-appearing white matter and grey matter, which are detailed below.

**Table 2:** Clinical scores in patients

	Raw means	*Z*-scores^a^
*n *=* *42 patients		
EDSS, median (range)	1.5 (0–3)	–
9-Hole Peg Test, s	20.4 ± 2.5	–
Timed 25-Foot Walk Test, s	4.4 ± 0.6	–
PASAT	−0.23 ± 1.2	−0.23 ± 1.2
SDMT	57.3 ± 9.6	−0.9 ± 1.1
BVMT-R	25.1 ± 5.9	−0.47 ± 1.1
CVLT-II	60.3 ± 9.2	1.1 ± 1.1

Data are presented as mean ± standard deviation, unless otherwise indicated. BVMT-R = brief visuospatial memory test-revised; CVLT-II = Californian verbal learning test II; EDSS = the Expanded Disability Status Scale; PASAT = paced auditory serial addition test; SDMT = symbol digit modalities test.

a *Z*-scores calculated from normative values displayed in the National Multiple Sclerosis Society Task Force database and from the normative data provided by the Brief International Cognitive Assessment for MS initiative.

**Table 3:** Statistically significant differences between patients and healthy controls in NDI, ODI and TSC across different white matter and grey matter areas

	Unstandardized coefficient (B)	Bootstrap sample CIs 99%	Effect size partial eta squared (η^2^_p_)	*P*-value
**NODDI (*n *=* *40 patients; *n *=* *16 healthy controls)**				
**NDI**				
Normal-appearing white matter				
Corpus callosum	−0.03	−0.06 to −0.006	0.12	0.004
Grey matter				
Right primary visual cortex	−0.02	−0.04 to −0.001	0.1	0.010
Left associative areas of occipital lobe	−0.016	−0.03 to −0.002	0.08	0.005
Left cognitive areas of parietal lobe	−0.014	−0.03 to −0.0007	0.002	0.009
Left superior-lateral temporal lobe	−0.012	−0.02 to −0.0006	0.09	0.009
**ODI**				
Normal-appearing white matter				
White matter (all)	0.008	0.002 to 0.02	0.08	0.003
Corpus callosum	0.008	0.00007 to 0.016	0.07	0.010
White matter occipital	0.02	0.006 to 0.001	0.09	0.003
White matter frontal	0.008	0.0007 to 0.016	0.06	0.007
White matter temporal	0.02	0.006 to 0.02	0.17	<0.0001
Grey matter				
Left middle frontal gyrus	−0.012	−0.02 to −0.0007	0.09	0.004
Left superior-lateral temporal lobe	−0.008	−0.01 to −0.0005	0.08	0.006
Right cognitive areas of parietal lobe	−0.01	−0.02 to −0.0003	0.12	0.009
Right operculum	−0.02	−0.04 to −0.001	0.01	0.010

**^23^Na MRI (*n *=* *34 patients; *n *=* *13 healthy controls)**				
**TSC**				
Normal-appearing white matter				
Corpus callosum	3.5	0.25 to 6.32	0.16	0.004
Grey matter				
Left middle frontal gyrus	5.81	0.51 to 11.21	0.15	0.006
Left limbic lobe	2.8	0.25 to 5.22	0.16	0.005
Right orbitofrontal cortex	2.40	0.11 to 4.88	0.14	0.009

Results from linear regression models are adjusted for age and sex. For grey matter areas, local volumes were included in the model if significantly different between patients and controls. Partial eta squared for the effect size of the variable ‘group’ defined as patients or controls.

### NODDI and total sodium concentration in white matter

**Figure 2:**
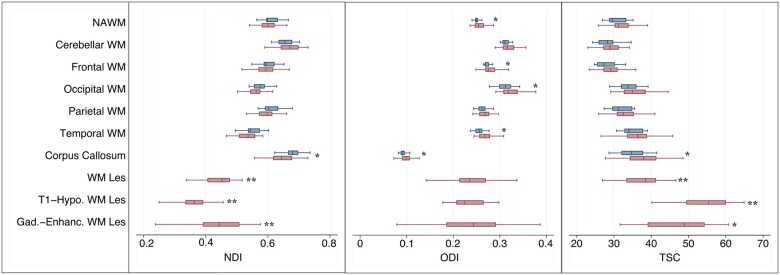
**NDI, ODI and TSC in the normal-appearing white matter and white matter lesions of patients and healthy controls.** Linear regression, corrected for age and sex for comparisons between patients (red box plots) and healthy controls (blue box plots); paired *t*-test for differences in NDI, ODI and TSC between white matter lesions and normal-appearing white matter. Gad. Enhanc. = gadolinium enhancing; Hypo. = hypointense; Les = lesion; NAWM = normal-appearing white matter; WM = white matter. *P < 0.01; ** *P < *0.0001.

**Figure 3:**
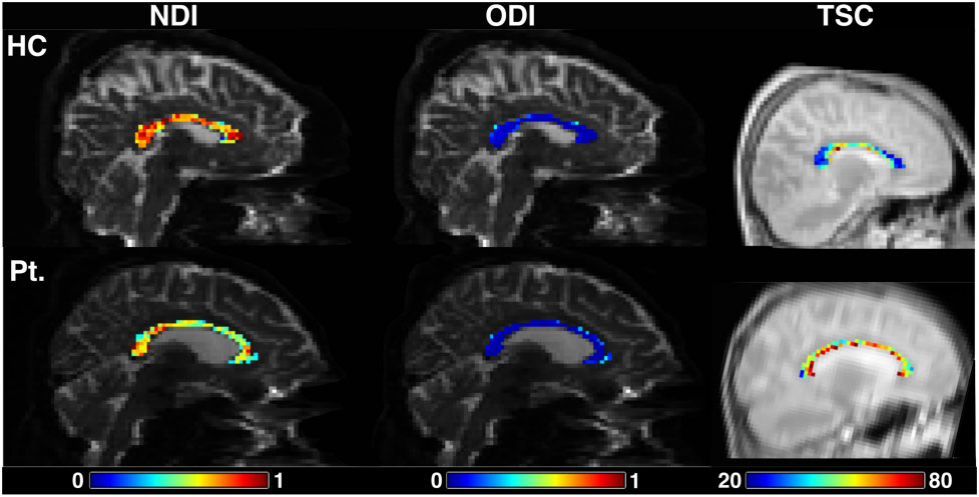
**NDI, ODI and TSC in the corpus callosum of a healthy control (*top*) and a patient (*bottom*).** HC = healthy control; Pt. = patient. TSC values are millimolar.

Compared with healthy controls, patients showed lower NDI in the corpus callosum. ODI was higher in the normal-appearing white matter of patients compared with healthy controls. In particular, ODI was increased bilaterally in occipital, frontal and temporal white matter as well as in the corpus callosum. TSC was higher in the corpus callosum of patients than healthy controls (Table 3, Figs 2 and 3).

T_2_-hyperintense white matter lesions showed higher TSC (B = 7.8; CI 4.8 to 10.6, *P*** ***<*** **0.0001) and lower NDI (B = −0.16; CI −0.18 to −0.14, *P*** ***<*** **0.0001) compared with normal-appearing white matter. A lower lesional NDI was associated with a higher lesional TSC (B = −0.05, CI −0.01 to −0.0006, *P* *<* 0.0001). T_1_-hypointense white matter lesions had higher TSC (14 patients; B = 25.41; CI 18.72 to 32.10, *P* *<* 0.0001), lower NDI (21 patients; B = −0.21; CI −0.25 to −0.18, *P* *<* 0.0001), and lower ODI (B = −0.02; CI −0.04 to −0.004, *P*** ***=*** **0.002) compared with normal-appearing white matter. Gadolinium-enhancing lesions had higher TSC (eight patients; B = 16.5; CI 3.8 to 29.10, *P*** ***=*** **0.002) and lower NDI (13 patients; B = −0.16; CI −0.22 to −0.09, *P*** ***<*** **0.0001) compared with normal-appearing white matter (Fig. 2).

### NODDI and total sodium concentration in grey matter

**Figure 4:**
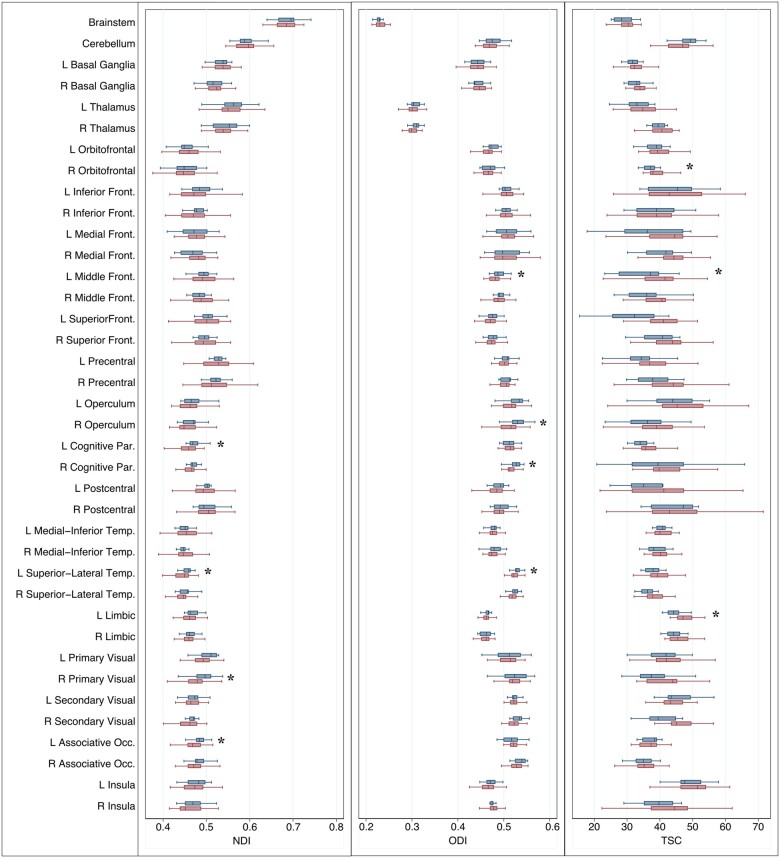
**NDI, ODI and TSC in the grey matter of patients and healthy controls.** *Linear regression, corrected for age and sex and local volumes, if found significantly different between patients (red box plots) and controls (blue box plots). Front. = frontal; L = Left; Occ. = occipital; Par. = parietal; R = Right; Temp. = temporal; *P < *0.01.

Compared with healthy controls, patients showed lower NDI in the right primary visual cortex, left occipital associative and parietal cognitive areas and left superior-lateral temporal lobe. The ODI was significantly decreased in the left superior-lateral temporal lobe, the left frontal middle gyrus and the right parietal cognitive areas. In patients, TSC was higher in the left frontal middle gyrus, left limbic lobe and right orbitofrontal cortex compared with healthy controls (Table 3 and Fig. 4).

### NODDI and total sodium concentration associations with lesion parameters

In patients, NDI, ODI and TSC found altered in white matter and grey matter areas did not correlate with T_1_-hypointense and T_2_-hyperintense white matter lesion volumes. As only eight patients had gadolinium-enhancing lesions, we did not assess the associations with the volume of this subgroup of lesions.

**Figure 5:**
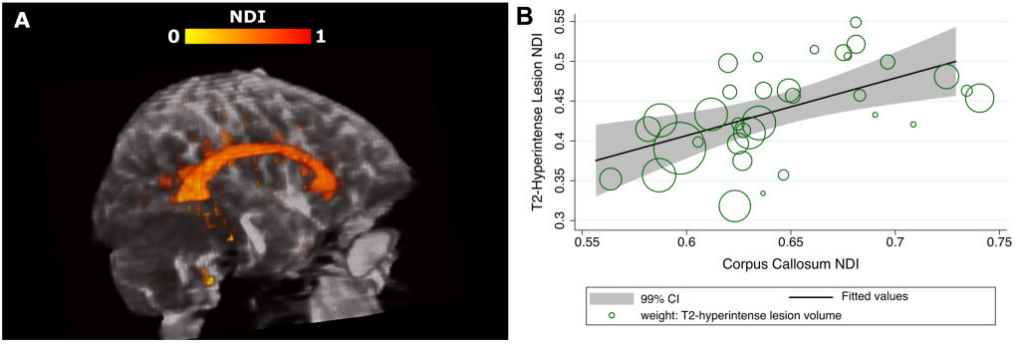
**Relationships between the NDI in the T_2_-hyperintense lesions and corpus callosum.** (**A**) Example of NDI in the corpus callosum and T_2_-hyperintense lesions of a patient. (**B**) Relationship between the NDI in the T_2_-hyperintense lesions and corpus callosum (markers are weighted by the T_2_-hyperintense lesion volume).

A lower NDI in the corpus callosum was associated with a lower NDI on the T_2_-hyperintense white matter lesions, even after adjustment for T_2_-hyperintense white matter lesion volume (B = 0.51, CI 0.27 to 0.84, *P*** ***<*** **0.0001) (Fig. 5).

### NODDI and total sodium concentration associations with clinical parameters

Brain parenchymal fraction, T_2_-hyperintense and T_1_-hypointense lesion volumes did not correlate with clinical variables.

A longer Timed 25-Foot Walk Test was associated with higher ODI in the corpus callosum (B = 0.01, 99% CI = 0.0001 to 0.02, *P*** ***=*** **0.009, R^2^ = 0.17). Neither the brain parenchymal fraction nor the T_2_-hyperintense lesion volume were significantly correlated with the Timed 25-Foot Walk Test (*P*** ***=*** **0.77 and *P*** ***=*** **0.4, respectively). When they were added as covariates, brain parenchymal fraction did not influence the significance of the correlation between the Timed 25-Foot Walk Test and corpus callosum ODI (ODI-B = 0.01, *P*** ***=*** **0.009, R^2^ = 0.26) whilst the T_2_-hyperintense lesion volume had a modest effect on the significance of this correlation (ODI-B = 0.01, *P*** ***=*** **0.013 R^2^ = 0.18). The ODI in the normal-appearing white matter did not correlate with any clinical outcomes. The NDI and TSC in the corpus callosum did not correlate with any clinical outcome. Higher disability, measured by EDSS, correlated with higher TSC in the left frontal middle gyrus (*r*** **=** **0.5, *P*** ***=*** **0.005).

We found borderline significance (0.01 < *P*-value < 0.05) for associations between NDI, ODI and TSC in some cortical areas and the 9-Hole Peg Test, CVLT-II, SDMT, and BVMT-R. These are reported in Supplementary Table 2. The rest of the NDI, ODI and TSC found altered in patients’ cortical areas (Table 3) did not correlate with clinical outcomes.

## Discussion

In this study, using quantitative MRI, we have demonstrated that CIS and early multiple sclerosis patients have microstructural and metabolic alterations in the brain not detectable by conventional MRI. Moreover, our novel multi-parametric approach shows early signs of axonal damage in the corpus callosum and sheds light on possible pathobiological mechanisms underlying this process.

### Fibre disorganization in normal-appearing white matter and axonal loss in white matter lesions

In the normal-appearing white matter of patients, we found widespread increases in ODI, particularly affecting the frontal, temporal and occipital lobes. We believe this could reflect inflammation and early fibre disorganization.

In multiple sclerosis, diffuse inflammation with lymphocytic infiltration and microglial activation can be observed in the normal-appearing white matter.^2^ These tissue changes are not seen with conventional MRI, as small alterations in the blood–brain barrier permeability do not permit the extravasation of gadolinium into the interstitium.^54^ As the presence of an activated microglial status may increase ODI,^55^ we hypothesize that the increased occupancy in the extra-neurite space related to inflammation could have driven the higher ODI in the normal-appearing white matter of our cohort.

Besides inflammation, axonal loss also dominates the pathology of normal-appearing white matter in multiple sclerosis, which may be a sequela of direct inflammation and, also, secondary Wallerian degeneration from white matter plaques.^2^ Previous NODDI studies^12–15^ enrolled established multiple sclerosis patients, who, compared with our early cohort, may have had more advanced neuroaxonal injury resulting in a consistent finding of decreased NDI in the normal-appearing white matter that we did not find. ODI, instead, has shown variable behaviour: it has been either higher in multiple sclerosis than healthy controls^14^^,^^56^ or both higher and lower, depending on the area examined,^15^ or unaltered.^12^^,^^13^ We believe the discrepancies of these previous studies may result from different degrees of axonal loss and inflammation in the tissue. In our study, normal-appearing white matter inflammatory processes may have been prevalent and caused initial degenerative changes in the axonal structure resulting in an increased fanning and/or dispersion of the fibres (i.e. high ODI) without yet a significant decrease in axonal density (i.e. low NDI). Longitudinal studies will help to understand how NODDI metrics can mirror pathological changes in the disease evolution.

In lesional white matter (i.e. T_2_-hyperintense lesions), we confirmed findings from previous multiple sclerosis studies^13^^,^^14^^,^^20–24^^,^^57^: lesions exhibit lower NDI and higher TSC compared with normal-appearing white matter, particularly in the T_1_-hypointense lesions. Interestingly, low lesional NDI correlated with high lesional TSC supporting the hypothesis that TSC and NDI together can be potential biomarkers of axonal damage as a consequence of demyelination and inflammation. For gadolinium-enhancing lesions in our study, NDI and TSC showed similar directional behaviour to T_1_ black holes, compared with normal-appearing white matter, i.e. lower NDI and higher TSC. This could indicate both acute axonal loss as well as oedema.^58^ In the T_1_-hypointense lesions ODI was low, supporting the hypothesis that in the presence of marked neuroaxonal loss, this index is low as the dispersion is estimated from relatively few axons.^10^^,^^14^

We performed additional *post hoc* analyses comparing isotropic volume fraction between T_2_-hyperintense lesions, T_1_-hypointense lesions, and normal-appearing white matter to investigate lesion characteristics. Isotropic volume fraction was higher in T_2_-hyperintense lesions (0.13 ± 0.04) and in T_1_-hypointense lesions (0.1 ± 0.08) than in normal-appearing white matter (0.09 ± 0.01) but only reaching significance for T_2_-hyperintense lesions (*P*** ***<*** **0.0001). This is somewhat consistent with the notion of increased water content in the lesions compared with normal-appearing white matter. However, isotropic volume fraction is known to be a highly variable metric and easily influenced by noise factors,^8^^,^^13^^,^^33^ hence these findings should be interpreted with caution.

### Early signs of axonal damage in the corpus callosum

NDI correlates primarily with axonal density and also with their degree of myelination.^10^^,^^59^ Therefore, the low NDI in the corpus callosum may reflect early axonal degeneration, particularly at the expense of large myelinated axons. The high corpus callosal ODI may not only be related to inflammation, as discussed before, but also be consequent to this reduced density of large axons. In a highly coherent structure, which, in health, has very low ODI, the orientation dispersion of the surviving axons can increase with neuroaxonal degeneration.

Our ^23^Na MRI findings of higher TSC in the corpus callosum support the hypothesis of early axonal damage within this structure and are consistent with a previous study in established multiple sclerosis.^23^ Demyelination and inflammation drive neuroaxonal damage through a cascade of events marked by an increased sodium influx in the axolemma due to at least two mechanisms. Firstly macrophages attack can damage mitochondria with consequent energy failure and sodium influx.^18^^,^^60^ In addition, demyelination can lead to a redistribution of ^23^Na channels along the axons to maintain ionic potentials.^16^ The consequences of these processes would be toxic calcium influx, which leads to neuroaxonal death.^19^ The coexistence of low NDI and high ODI suggests that TSC may be increased consequent to both increased extracellular volume, due to axonal loss, and increased intracellular concentration, due to axonal metabolic dysfunction.

Our study showed lower NDI of the corpus callosum correlated with lower NDI in white matter lesions, suggesting that retrograde neurodegeneration from the transected axons of the affected white matter could influence axonal integrity in the corpus callosum.^61^ This structure may be particularly susceptible to Wallerian degeneration having a high density of fibres, all in the same direction, that could degenerate secondarily from the same distant demyelinating lesion.

Overall, our novel results show the presence of axonal damage, not captured by conventional MRI techniques, in the corpus callosum of patients within 3 months of symptom onset.

The presence of demyelinating plaques in the corpus callosum is a distinctive feature of multiple sclerosis, shared by few other conditions,^62^ and disease-related corpus callosal atrophy has been documented in post-mortem cases.^63^ Therefore, researchers have investigated changes in the corpus callosum of CIS patients. Using diffusion tensor imaging, they showed increased mean diffusivity^64^ and reduced fractional anisotropy^27^^,^^65^ in this area. However, diffusion tensor imaging indices are less specific than NODDI at differentiating neurodegeneration from other microstructural substrates and a decreased fractional anisotropy may be just related to an increased ODI.^10^^,^^66^ Other studies using diffusion tensor imaging parameters of axial and radial diffusivity demonstrated increased axial diffusivity,^67^^,^^68^ an index that has been associated with axonal damage.^69^ However, the lesions were excluded only with probability maps^68^ or not excluded at all^67^ from the analysis, so they could have influenced their findings. Two studies, from the same group, assessed possible metabolic alterations in the corpus callosum using MRS.^6^^,^^7^ In the first study, the authors concluded that only demyelination, and not neurodegeneration, occurred in the corpus callosum because the *N*-acetylaspartate/creatine ratio was not altered. However, MRI spectroscopy results were reported as metabolite ratios and this stability could have been caused by a similar change in the two metabolites. In the latter study, they found decreased *N*-acetylaspartate, as a sign of axonal dysfunction, in the corpus callosum, but callosal lesions were included in the analysis.

### Metabolic and microstructural alterations in cortical grey matter

In grey matter, we found metabolic and microstructural alterations in several cortical regions. Previous studies in established multiple sclerosis did not find significant differences in the NDI and ODI of the whole grey matter between patients and healthy controls,^12^^,^^13^ but did in certain areas.^15^ This is not surprising as, compared with white matter, significant alterations in the whole cortex may be more difficult to detect given its non-homogenous microstructure: NDI and ODI can vary according to the specific myelo- and cyto-architecture of different cortical areas.^70^

Topographically, our patients, most of them with optic neuritis, had low NDI in the visual areas. The presence of retrograde axonal degeneration following the optic nerve damage has been described.^71^ This may suggest a possible relationship with damage to the anterior visual pathway that deserves further investigation in future studies.

Our findings of low ODI in certain grey matter areas could indicate complex tissue changes in the cortex. A recent study showed that ODI captures the cortical myeloarchitecture: it is high in the granular cortex, which possesses tangential myelinated fibre bands, and low in the agranular areas, such as the insula and cingulate cortex. Moreover, ODI showed a strong negative correlation with the cortical thickness.^70^ Since early patients can show dynamic changes in cortical morphology,^72^ we hypothesize that the low ODI here reflects complex cortical changes, such as the loss of tangential myelinated fibres.

We found higher TSC in some grey matter areas compared with healthy controls. TSC was found increased in established multiple sclerosis.^22–25^ In early multiple sclerosis and CIS, alterations due to microstructural pathology may still be only localized to a few areas and, over time, may become more diffuse. Moreover, functional and dynamic metabolic changes can also contribute to an increase in TSC.^42^

### Associations with clinical parameters

Our patients had, as expected, low disability, limiting the likelihood of detecting correlations with clinical outcomes. Nevertheless, even though we restricted our analysis to the areas where significant alterations were found, our results offer interesting insights.

First, we found that higher ODI in the corpus callosum correlated with a longer Timed 25-Foot Walk Test (*P*** ***=*** **0.009). As this area is the largest interconnecting fibre tract in the brain, it is probable that is involved in gait regulation.^73^ Second, although we performed ^23^Na MRI in a cohort of patients early after onset, we were able to confirm previous findings of associations between cortical TSC and physical disability.^23^^,^^25^ In our study, this was restricted to a specific gyrus. Nevertheless, it suggests that ^23^Na MRI can detect pathological abnormalities relevant to physical disability even in early patients.

Finally, as hypothesis-generating results (0.01 < *P*** ***<*** **0.05), we observed that lower ODI in grey matter areas involved in attention and language (i.e. the left middle frontal gyrus) and cognition (i.e. the right operculum) was associated with worse cognitive performance (Supplementary Table 2). This suggests that early changes in cortical morphology may affect cognition, as previously reported^72^ and support our hypothesis that low ODI may reflect complex changes such as the loss of of tangential myelinated fibres.

Interestingly, no significant clinical associations were found for brain parenchymal fraction or T_2_-hyperintense lesion volume, implying that NODDI and ^23^Na MRI may offer more sensitive and clinically relevant markers of tissue pathology in early multiple sclerosis than conventional MRI metrics.

### Limitations

We acknowledge several limitations of our study.

First, we did not recruit a large number of subjects and not all the subjects who underwent NODDI also completed the ^23^Na MRI protocol. However, being the first study on NODDI and ^23^Na MRI in very early patients and the first one combining the two techniques, we believe that the current sample size can justify the exploratory aims of this research. Furthermore, we chose a significance level of *P* ≤ 0.01 for robust regression estimates and we limited correlations with clinical outcomes only to areas showing significant differences in NDI, ODI and TSC between patients and controls.

Second, the discussion of our results for NODDI is based on previous histological findings in animal models and observational studies in neurological disorders, and for ^23^Na MRI, on previous observational studies in multiple sclerosis. We cannot directly demonstrate that these measures are sensitive to the different pathophysiological substrates. Only pathological studies can provide this evidence, and, for early patients, they are rare and performed in small and/or atypical cohorts.

Our study performed NODDI on a clinical scanner. Our clinical implementation requires echo times longer than ones achievable at ultra-high gradient strength, theoretically implying lower signal-to-noise levels. Nonetheless, previous work^8^^,^^31^ has demonstrated that the echo time used in this study is sufficient to produce image quality suitable for group comparisons. Another methodological consideration relates to the design of the diffusion MRI NODDI protocol. In this study, we used 45 gradient directions, against the 90 prescribed in the original NODDI publication,^8^ to optimize total scanning time. Previous results have shown that protocols comprising as low as 30 directions are sufficient to reliably characterize neurite density and dispersion.^8^

To investigate patients at onset, we assessed most of them in proximity to their first demyelinating episode. It is possible that this could have influenced our clinical outcomes.

Finally, the low ^23^Na sensitivity at 3 T means that we could derive only TSC, rather than intracellular and extracellular sodium concentrations. Nevertheless, using NODDI, we could identify areas of low NDI where increased extracellular volume due to axonal loss could have contributed to the increased TSC. High field scanners (e.g. 7 T) are required to disentangle the intracellular and extracellular component of the TSC changes^23^ and could be considered in future studies.^74^

## Conclusions

In summary, we found that CIS and multiple sclerosis patients at onset have diffuse axonal dispersion in normal-appearing white matter, potentially related to inflammation, whilst they exhibit alterations consistent with axonal pathology and dysfunction in the corpus callosum, a typical site for multiple sclerosis involvement, and in cortical areas. Our study suggests that the combined use of NODDI and ^23^Na MRI can detect and provide insights, *in vivo*, into early multiple sclerosis pathology. Our results report clinical associations with NODDI and ^23^Na imaging, not evident with more conventional measures of the brain or T_2_-hyperintense lesion volumes. Future longitudinal studies can assess whether these changes correlate with disability accumulation.

## Supplementary Material

awab043_Supplementary_Data

## Abbreviations

CIS: clinically isolated syndrome
NDI: neurite density index
NODDI: neurite orientation dispersion and density imaging
ODI: orientation dispersion index
TSC: total sodium concentration
