# Supplementary material for: Brain microstructural and metabolic alterations detected in vivo at onset of the first demyelinating event
Source: Brain. 2021 Apr 27;144(5):1409–21. doi: 10.1093/brain/awab043 (PMC8219367; doi:10.1093/brain/awab043)
Supplement: awab043_Supplementary_Data [file awab043_supplementary_data.pdf]

Supplementary Table 1. Cortical and white matter areas in which neurite density index, orientation dispersion index and total sodium concentration have been computed

Areas	Structures	Side
**Grey Matter**		
**Cerebellum Grey Matter**	Vermis, Lobes	L+R
**Thalamus**	Thalamus	L; R
**Limbic Lobe**	ACgG, MCgG, PCgG, PHG, Ent, Amigdala, Hippocampus	L; R
**Hippocampus**	Hippocampus	L; R
**Insula**	AIns, PIns	L; R
**Orbitofrontal cortex**	AOrG, POrG, MOrG, LOrG, GRe, SCA	L; R
**Medial Frontal cortex**	Medial Frontal cortex	L; R
**Middle Frontal gyrus**	Middle Frontal gyrus	L; R
**Superior Frontal gyrus**	SFG, MSFG	L; R
**Inferior Frontal gyrus**	OpIFG, OrIFG, TrIFG	L; R
**Precentral gyrus**	MPrG, PrG	L; R
**Operculum**	CO, FO, PO	L; R
**Postcentral gyrus**	PoG, MPoG	L; R
**Cognitive Areas of the Parietal lobe**	AnG, PCu, SMG, SPL	L; R
**Superior-lateral Temporal lobe**	MTG, TTG, STG, PP, PT	L; R
**Medial-inferior Temporal lobe**	FuG, ITG, TMP	L; R
**Primary Visual cortex**	Calc, Cun, OCP	L; R
**Lingual gyrus**	Lingual gyrus	L; R
**Associative Areas of the Occipital lobe**	IOG, MOG, SOG, OFuG	L; R
**White Matter**		
**Cerebellar white matter**		L+ R
**Insular white matter**		L+ R
**Frontal white matter**		L+R
**Parietal white matter**		L+ R
**Temporal white matter**		L+ R
**Occipital white matter**		L+ R
**Corpus Callosum**		**-**

Note: parcellations were based on the the Desikan-Killiany-Tourville protocol (Klein and Tourville, 2012)

Abbreviations: ACgG: anterior cingulate gyrus; MCgG: middle cingulate gyrus; PCgG: posterior cingulate gyrus; PHG: parahippocampal gyrus; Ent: entorhinal area; AIns: anterior insula; Pins: posterior insula; AOrG: anterior orbital gyrus; POrG: posterior orbital gyrus;

MOrG: medial orbital gyrus; LOrG: lateral orbital gyrus; GRe: gyrus rectus; SCA:sub-callosal area; SFG: superior frontal gyrus; MSFG: superior frontal gyrus medial segment; OpIFG: opercular part of the inferior frontal gyrus; OrIFG: orbital part of the inferior frontal gyrus; TrIFG: triangular part of the inferior frontal gyrus; MPrG: precentral gyrus medial segment; PrG: precentral gyrus; CO: central operculum; FO: frontal operculum; PO: parietal operculum; PoG: postcentral gyrus; MPoG: postcentral gyrus medial segment; AnG: angular gyrus; PCu: pre-cuneus; SMG: supramarginal area; SPL: superior parietal lobule; MTG: middle temporal gyrus; TTG: transverse temporal gyrus; STG: superior temporal gyrus; PP: planum polare: PT: planum temporale; FuG: fusiform gyrus; ITG: inferior temporal gyrus; TMP: temporal pole; Calc: calcarine cortex; Cun: cuneus; OCP: occipital pole; IOG: inferior occipital gyrus MOG: middle occipital gyrus; SOG: superior occipital gyrus; OFuG: occipital fusiform gyrus.

Supplementary Table 2. Borderline significant association (0.01<p-value<0.05) between clinical parameters and neurite density index, orientation dispersion index and total sodium concentration

Dependent Variable	Independent Variable	Unstandardized coefficient (B)	Confidence Interval 95%	p-value	R^2
9-hole peg test	Left limbic lobe TSC	0.52	0.12 to 0.93	0.012	0.19
SDMT (z-score)	Left frontal middle gyrus ODI	0.06	0.0004 to 0.01	0.038	0.11
CVLT-II (z-score)	Right primary visual cortex NDI	-0.09	-0.02 to -0.0004	0.039	0.12
BVMT-R (z-score)	Right operculum ODI	-0.01	-0.02 to -0.001	0.028	0.12
**With T2-hyprintese lesion volume as covariate**					
9-hole peg test	Left limbic lobe TSC	0.52	0.12 to 0.93	0.012	0.19
SDMT (z-score)	Left frontal middle gyrus ODI	0.06	0.0003 to 0.01	0.04	0.11
CVLT-II (z-score)	Right primary visual cortex NDI	-0.09	-0.02 to -0.0006	0.037	0.12
BVMT-R (z-score)	Right operculum ODI	-0.01	-0.02 to -0.0009	0.031	0.12
**With brain parenchymal fraction as covariate**					
9-hole peg test	Left limbic lobe TSC	0.52	0.12 to 0.93	0.012	0.19
SDMT (z-score)	Left frontal middle gyrus ODI	0.06	-0.000009 to 0.01	0.05	0.11
CVLT-II (z-score)	Right primary visual cortex NDI	-0.08	-0.02 to 0.0003	0.057	0.12
BVMT-R (z-score)	Right operculum ODI	-0.009	-0.02 to -0.00009	0.048	0.12

Note: results are from linear regression model

Abbreviations: SDMT: symbol digit modality test; CVLT-II: California Verbal Learning Test-II; BVMT-R: brief visuospatial memory test-revised
